# Development of a root canal treatment model in the rat

**DOI:** 10.1038/s41598-017-03628-6

**Published:** 2017-06-12

**Authors:** Naomichi Yoneda, Yuichiro Noiri, Saori Matsui, Katsutaka Kuremoto, Hazuki Maezono, Takuya Ishimoto, Takayoshi Nakano, Shigeyuki Ebisu, Mikako Hayashi

**Affiliations:** 1 Department of Restorative Dentistry and Endodontology, Osaka University Graduate School of Dentistry, 1-8 Yamadaoka, Suita, 565-0871 Japan; 2 Division of Cariology, Operative Dentistry and Endodontics, Department of Oral Health Science, Niigata University Graduate School of Medical and Dental Sciences, 2-5274, Gakkocho-dori, Chuo-ku, Niigata 951-8514 Japan; 3 Department of Biomaterials and Structural Materials Design, Osaka University Graduate School of Engineering, 1-2 Yamadaoka, Suita, 565-0871 Japan

## Abstract

Root canal treatment is performed to treat apical periodontitis, and various procedures and techniques are currently used. Although animal models have been used in the developmental research of root canal treatment, little of this research has used small animals such as rats, because of their small size. In this study, root canal treatment was performed on the rat mandibular first molar, which had four root canals, using a microscope, and the therapeutic effect was evaluated bacteriologically, radiologically and histopathologically. By performing root canal treatment, the level of bacteria in the mesial root of the treated teeth was reduced by 75% compared with the control. Additionally, the volume of the periapical lesions of the treated teeth as measured by micro-computed tomography decreased significantly 2 weeks after the root canal treatment when compared with the control. Histological evidence of healing was observed in the treatment group 8 weeks after root canal treatment. These results suggest that a root canal treatment model using rats can be used in developmental research for novel methods of root canal treatment.

## Introduction

Apical periodontitis is an inflammatory disease in the periapical area which results from infection of the dental pulp in the root canal system. Root canal treatment aims to remove bacteria from the root canal system, and dentists achieve this through mechanical removal of infected dentin and chemical removal of remnant bacteria in the root canal. This treatment is based on scientific evidence that germ-free mice do not develop apical periodontitis, and bacterial infection of the pulp chamber or root canal can cause apical periodontitis^1^. The success rate of initial root canal treatment is found to be lower than that of pulpectomy, which is the treatment for inflamed dental pulps without bacterial infections^2^. It is difficult to eliminate bacteria from root canal systems, not only because of their complex anatomical structure, but also because bacteria are embedded in biofilms. Biofilms are formed not only in the root canal system but also on the outer root surface around the apical foramen^3–5^, where they are known as extraradicular biofilms. Extraradicular biofilms lower the success rate of root canal treatment, making apical periodontitis refractory^6^.

The rate of bacteria removal by root canal treatment is not known. Root canal treatment is thought to be an aseptic treatment, but the validity of this claim is unknown.

Recently, techniques such as dental microscopy, cone-beam computed tomography (CT) and micro-excavation have been introduced into clinical dental practice. These techniques have greatly improved the accuracy of preoperative diagnosis and treatment techniques^7–9^, making it possible to preserve teeth which would previously have been extracted. However, refractory apical periodontitis has still not been eradicated, and the development of new drugs or treatment methodologies is essential.

Extraradicular biofilm that is present on the apical cementum and over-filled root canals has been implicated in the development of persistent apical periodontitis^10^. We formed extraradicular biofilm experimentally in rats and confirmed its influence on the volume of periapical lesions using high resolution micro-CT^11^. Our novel approach to the control and inhibition of biofilm-forming bacteria involved observing the activity of N-acyl homoserine lactones which take part in the quorum-sensing system for bacterial cell-to-cell communication. We found that three analogues inhibited the biofilm formation of *Porphyromonas gingivalis* ^12^, and that antimicrobial azithromycin controlled *P. gingivalis* biofilm at sub-minimum inhibitory concentration levels^13^.

Although research has elucidated mechanisms and methods for using various medicaments to heal apical periodontitis, it is necessary to establish an evaluation system to confirm the efficacy of these methods by using a laboratory animal model before they are introduced to clinical practice. Various experiment models using small animals which reproduce the condition of a human patient have been used in the study of marginal periodontitis, and a new therapeutic drug and methodology have been developed^14–17^. However, while a number of small animal models have been developed to study the pathology of apical periodontitis^18–20^, there are no existing models for the study of root canal treatment. This is because rodent teeth have been considered to be too small for root canal treatment, and they have no teeth that can easily undergo root canal treatment such as the human single-rooted permanent incisor or premolar. Consequently, there are no studies worldwide reporting successful treatments of infected root canals in small animals. However, innovations in the therapeutic apparatus of endodontic treatment, such as the dental microscope and the micro-excavator, now allow correct diagnosis and precise root canal treatment, so that even a small rodent can undergo root canal treatment. Additionally, accurate three-dimensional analysis of periapical lesions in small animals has been enabled by high resolution micro-CT, along with improved evaluation of the efficacy of the treatment. Therefore, in this study, we aimed to develop a small animal root canal treatment model by treating infected root canals of rats using the latest treatment apparatus under microscopic observation. We then analysed the periapical lesions three-dimensionally using micro-CT over time to evaluate the postoperative progress of the lesions.

## Materials and Methods

### Ethics statement

This study was approved by the Animal Care and Use Committees of the Osaka University Graduate Schools of Dentistry and Engineering (Permit Nos 26-016-0 and 26-1-0). All animal experiments were carried out in accordance with the Guidelines for Animal Experiments of Osaka University, and surgical procedures were performed under sodium pentobarbital anaesthesia, and all efforts were made to minimise the animals’ suffering.

### Animals

Four 4-week-old and nine 10-week-old male Wistar rats (Clea Japan, Inc., Tokyo, Japan) were used. The animals were maintained in the animal facility of the Osaka University Graduate School of Dentistry with a 12-h light/12-h dark cycle. Food and water were freely available.

### Root length/canal width measurement

Four 4-week-old male Wistar rats underwent general anaesthesia with an intraperitoneal injection. The mandibular first molar was scanned with micro-CT (R_mCT2, Rigaku, Tokyo, Japan) at settings of 90 kV and 160 µA every week from 4 to 14 weeks. A total of 500 consecutive tomographic slices, each with a thickness of 20 µm, were acquired. SimpleViewer software (Rigaku) was used for image analysis. After the axes were standardised, the length of the mesial root and the width of the mesial root canal (the widest dimension of the root canal at a point 1 mm apical to the pulpal floor) were measured.

### Root canal treatment in rats

**Figure 1:**
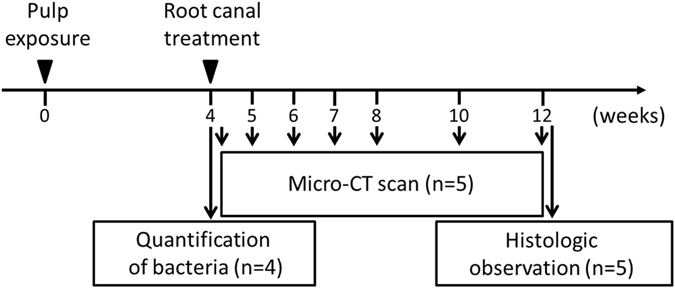
Experimental protocol. Root canal treatment was performed 4 weeks after pulp exposure and was evaluated by micro-CT scanning, quantification of bacteria, and histological observation.

Nine 10-week-old male Wistar rats were used for this experiment, and the experiment design is shown in Fig. 1. All surgical procedures were performed under microscopic observation (Stemi DV4 SPOT, Carl Zeiss, Oberkochen, Germany). Periapical lesions were induced by exposing the pulp of mandibular first molars using a #1/2 (ISO 006) round bur (Dentsply Maillefer, Ballaigues, Switzerland) and electric engine (VIVAMATE G5, NSK, Tochigi, Japan) and leaving them open to the oral environment for 4 weeks^21^. Maxillary first molars in contact with the experimental teeth were removed at the same time as the pulp exposure of the mandibular first molars to prevent tooth fracture. The right mandibular first molars underwent root canal treatment as the treatment group, and the left mandibular first molars were left untreated as a control group.

**Figure 2:**
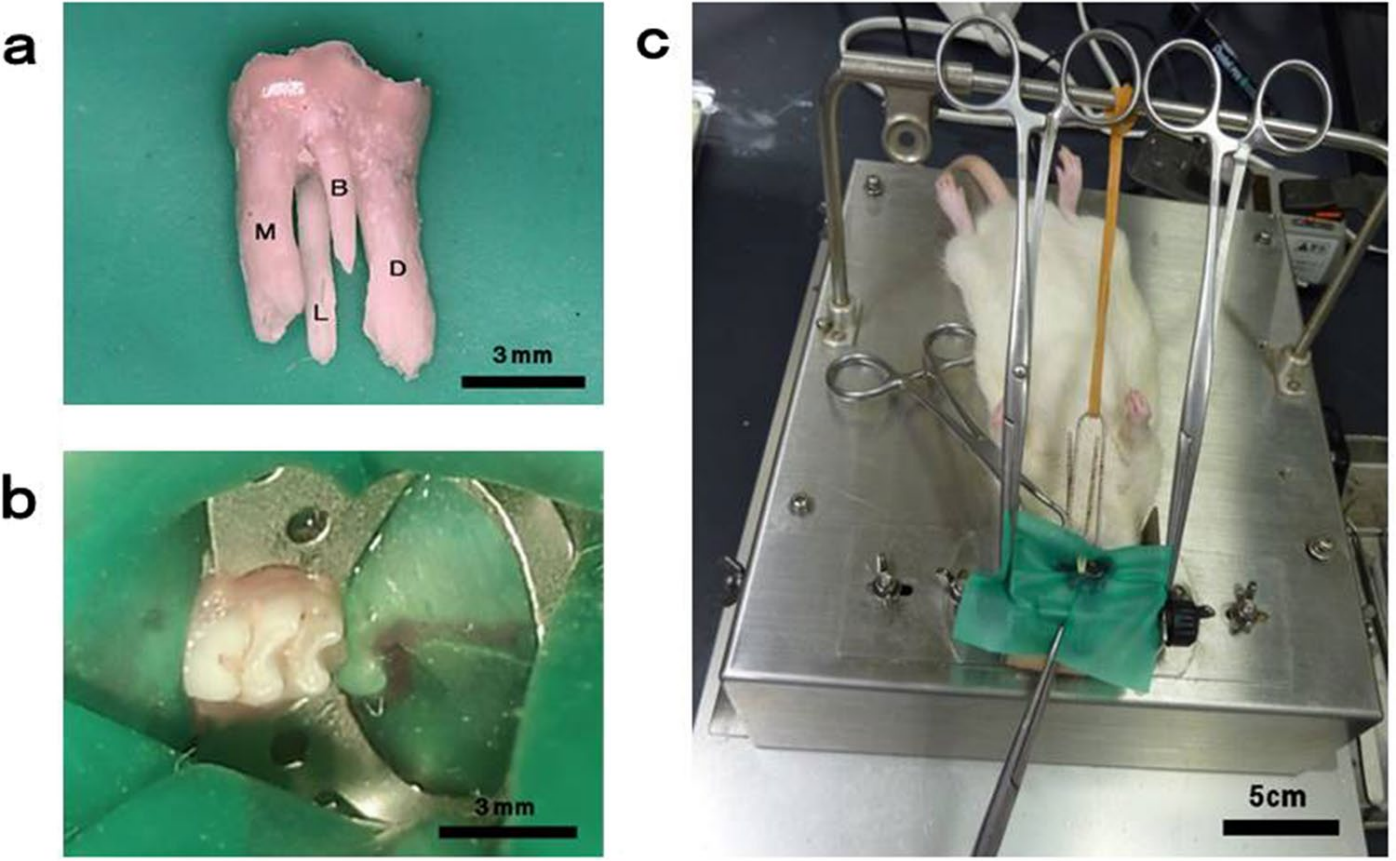
Rubber-dam isolation of the rat mandibular molar. (**a**) Rat mandibular first molar with four roots and a crown with a mesiodistal diameter of approximately 3 mm. (**b**,**c**) Isolation of the tooth with a custom-made rubber-dam clamp.

For the treatment group, the tooth was isolated with a custom-made rubber dam clamp (YDM, Tokyo, Japan) and rubber dam sheet (Heraeus Kulzer, South Bend, USA) (Fig. 2b,c). The gap between the tooth and the rubber dam was blocked using flowable composite resin (MI FLOW, GC, Tokyo, Japan). An aseptic state was established by cleaning the test tooth with 70% ethanol. A #1/2 round bur was used to open the pulp chamber and remove necrotic coronal pulp, and a micro-excavator (OK Micro-exca, Seto, Ibaraki, Japan) was used to remove the infected tooth substance of the pulpal floor and the orifice of the root canal. Root canal enlargement was performed to the level of 1.0 as indicated by an electrical root canal meter (Root ZX, J Morita, Tokyo, Japan) using K-files (Dentsply Maillefer, Ballaigues, Switzerland) up to a #20 file for the mesial and distal roots and a #15 file for the buccal and lingual roots. A nickel-titanium (Ni-Ti) rotary file (Race, FKG, La Chaux-de-Fonds, Switzerland) was then used to prepare the root canal. The mesial and distal roots were prepared with a 4% taper, and the buccal and lingual roots were prepared with a 2% taper. Root canals were irrigated with 0.5 ml of 2.5% sodium hypochlorite (Neo Dental Chemical Products, Tokyo, Japan) using 30-gauge needles (NaviTip, Ultradent Products, South Jordan, UT), the flow rate was 1 ml/min. Then canals were dried using sterilised paper points (VDW, Munich, Germany), and filled with gutta-percha points (SybronEndo, Orange, CA, USA) and root canal sealer (RealSeal SE, SybronEndo) using the single point method. After processing with a bonding system (Clearfil Bond SE ONE, Kuraray Noritake Dental, Tokyo, Japan), the pulp chamber was filled with flowable composite resin (MI FLOW, GC).

### Three-dimensional measurement of the periapical lesion volume

At 4, 5, 6, 7, 8, 10, and 12 weeks after pulp exposure, the induced periapical lesions were scanned with a micro-CT scanner (Rigaku). Five rats were tested at each time point, and each rat had a control site and an experimental site. After scanning, the image data were reconstructed using the Three-Dimensional Reconstruction Imaging for Bone (TRI/3D-BON) system (Ratoc System Engineering, Tokyo, Japan) and the volume of the periapical lesion at the mesial root was measured as previously described^11^.

### Quantification of bacteria in the root canal

Immediately following the root canal treatment, the rats were sacrificed and the mandibular first molars on both sides were extracted. The teeth were cut at the mesial root furcation and bacteria were removed from the root surface by curetting with a sterilised spoon excavator (YDM). The mesial root was then frozen in liquid nitrogen and crushed using an SK mill (Tokken, Chiba, Japan). Following a previously described method^11^, DNA extraction was performed on a powdered sample using the InstaGene Matrix (Bio-Rad Laboratories, Hercules, CA, USA) according to the manufacturer’s instructions. Assays were performed with a 20 µl solution containing 1 µl of DNA extract (Applied Biosystems Power SYBR Green PCR Master Mix; Life Technologies, Grand Island, NY, USA) and bacterial universal primers 357 F and 907R^22^ (0.5 µl each), prepared in parallel reaction mixtures for each target sequence. The thermal cycling conditions for the Applied Biosystems 7500 Fast Real-Time PCR system (Life Technologies) were 95 °C for 10 min, 40 cycles at 95 °C for 15 s, and 65 °C for 1 min, with collection of the fluorescence signal at the end of each cycle. Melting-curve analysis consisted of a denaturation step at 95 °C for 15 s and a temperature reduction to 60 °C for 1 min followed by a temperature increase to 95 °C at a rate of 1%, with continuous fluorescence reading. Data were acquired and analysed using Applied Biosystems 7500 system SDS v2.0.2 software (Life Technologies). *Enterococcus faecalis* SS497 was used as a standard curve.

### Histological observation

The rats were sacrificed 12 weeks after pulp exposure, which is 8 weeks after treatment (n = 5). Mandibular samples containing the first molars were dissected, fixed in 4% paraformaldehyde and 0.1% glutaraldehyde for 12 h at 4 °C, and decalcified in 10% EDTA containing 15% glycerol at 4 °C. After preparation of 7 µm thick serial sections, some sections were stained with haematoxylin and eosin (HE) and a modified Brown and Brenn method^23^, and observed under a light microscope (Optiphot-2; Nikon Corporation, Tokyo, Japan).

### Statistical analysis

Welch’s *t*-test was used to check the statistical significance of changes in the periapical lesion volume between the treatment group and the control group, and Kruskal-Wallis test was used for significant difference to 4-week volume of the same group with a critical rate of 5%. Additionally, for bacterial quantification, the Steel-Dwass test was used with a critical rate of 5%.

## Results

### Measurement of root length and root canal width

**Figure 3:**
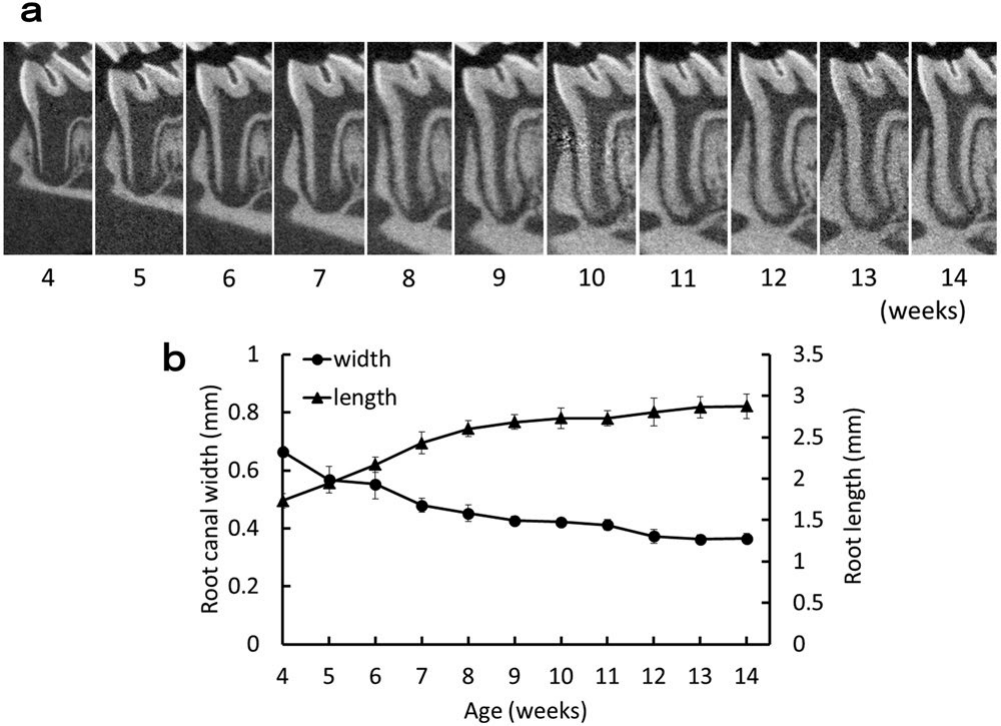
Age-related change in the mesial root of the mandibular first molar. (**a**) Representative micro-CT images of the mesial root of the mandibular first molar at each week of age. (**b**) Age-related change in root length and root canal width. Root length was measured from the pulpal floor to the root apex; the root canal width was measured at 1 mm apically from the pulpal floor. Data represent the means of four sample measurements; error bars indicate standard deviations.

The mean root length of the mesial root was 1.7 mm at 4 weeks of age, increasing to 2.6 mm at 8 weeks. The mean root canal width was 0.66 mm at 4 weeks of age, but decreased to 0.37 mm at 12 weeks (Fig. 3b). These results confirm that tooth root development in the Wistar rat mandibular first molar is complete at 8 weeks of age. We therefore used 10-week-old rats in this experiment because they satisfied two conditions: (1) definite tooth root completion; and (2) proper root canal diameter for root canal treatment.

### Three-dimensional measurement of the periapical lesion volume

**Figure 4:**
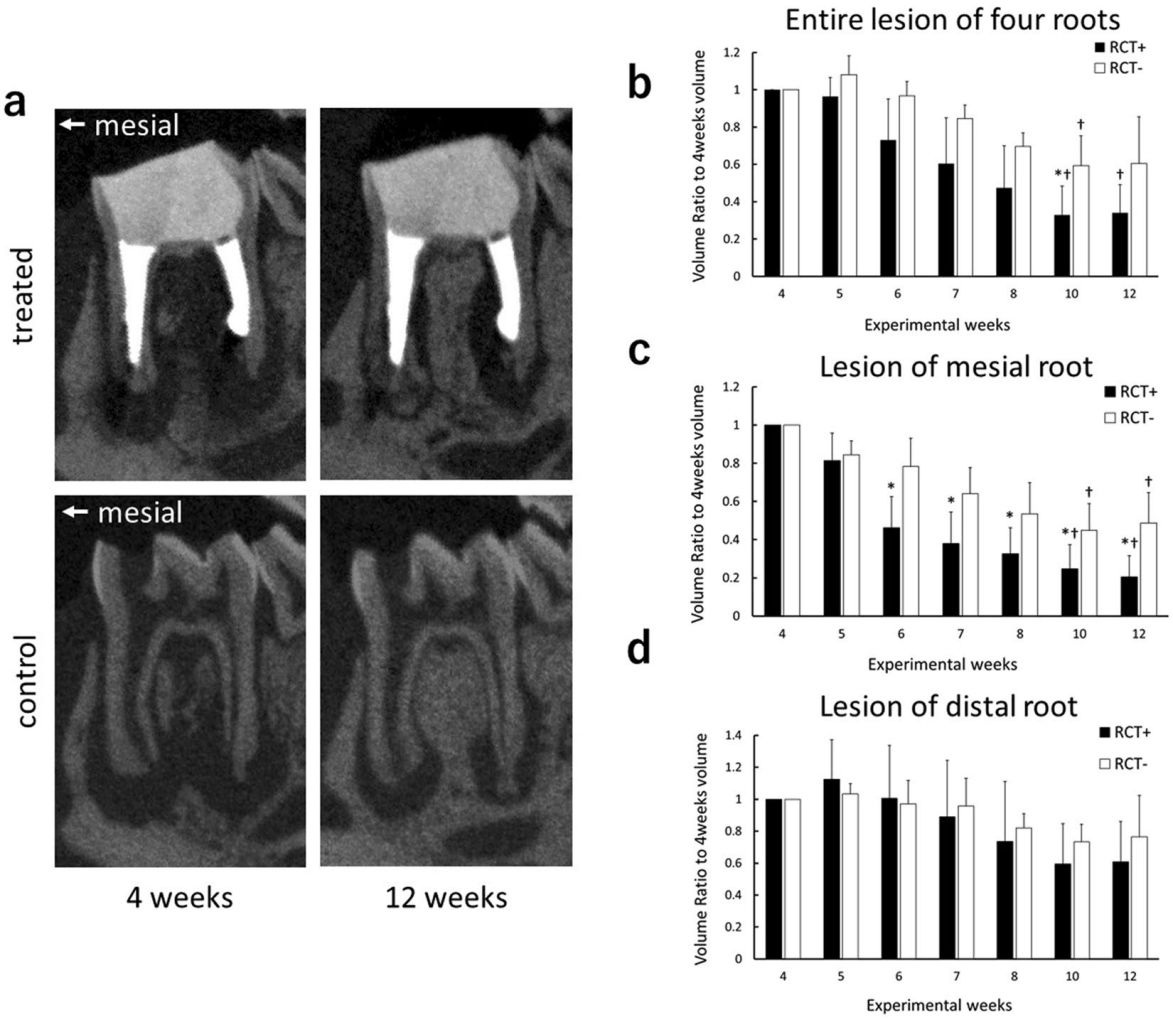
Micro-CT analysis of periapical lesions after root canal treatment in rat. (**a**) Representative micro-CT images of experimental teeth in the treatment group, and the control group, at the 4-week and 12-week time points. (**b**–**d**) Comparison of changes in the volume of periapical lesions (*P < 0.05 indicates significant differences compared with the control group at the same time point, Welch’s *t*-test; ^†^ *P* < 0.05 indicates significant differences compared with the 4-week data of the same group, Kruskal-Wallis test). Data represent the means of five sample measurements; error bars indicate standard deviations. The volume of the periapical lesions of the mesial root for the treatment group were significantly lower than that of the control group at each time point after 6 weeks following pulp exposure.

The mean volume of periapical lesions at 4 weeks after exposure was 8.04 mm^3^ in the treatment group and 7.46 mm^3^ in the control group, and no statistically significant difference was found between the two groups. At 10 weeks after exposure (6 weeks after root canal treatment), the volume of the entire periapical lesion of the treatment group (2.48 mm^3^) was significantly smaller than that of the control group (4.78 mm^3^). The periapical lesion volume in the treatment group at 10 weeks was 33% of the volume at 4 weeks after exposure. In the control group, the minimum periapical lesion volume at 10 weeks was 59% of the volume at 4 weeks after exposure (Fig. 4b).

The mean volume of mesial periapical lesions at 4 weeks after exposure was 4.54 mm^3^ in the treatment group and 4.85 mm^3^ in the control group. No statistically significant difference was found between the two groups. The mesial periapical lesion volume in the treatment group was significantly smaller than that of the control group at each time point after 6 weeks following exposure. In the treatment group, the mesial periapical lesion volume became 20% of the volume at 4 weeks after exposure. Also, control group showed the minimum volume of 44% compared to the data of 4 weeks after exposure (Fig. 4c).

The mean volume of distal periapical lesions at 4 weeks after the exposure was 4.69 mm^3^ in the treatment group and 4.55 mm^3^ in the control group. No significant difference was observed between the two groups. There was no significant difference in distal periapical lesion volume between the treatment group and the control group at any time point. The distal periapical lesion volume in the treatment group tended to decrease to 59% of the volume measured at 4 weeks after pulp exposure, and the control group tended to decrease to 73% (Fig. 4d).

### Quantification of bacteria in the root canal

**Figure 5:**
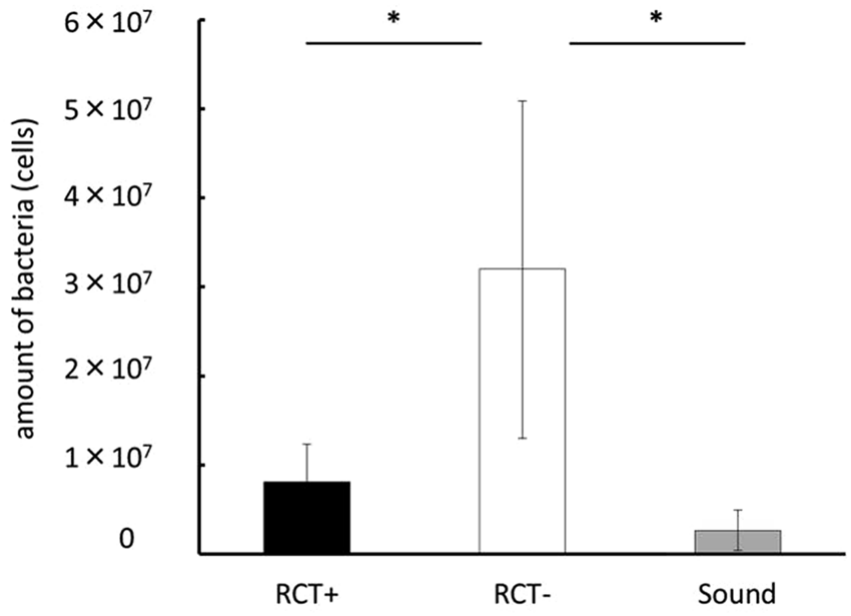
Quantification of bacteria derived from the mesial root immediately after root canal treatment using real-time PCR (*P < 0.05 indicates significant differences between the two groups, Steel-Dwass test). Data represent the means of four sample measurements; error bars indicate standard deviations.

The bacterial level derived from the mesial root immediately after root canal treatment of the infected root is shown in Fig. 5. The number of bacteria in the mesial root was significantly lower for the treatment group (0.8 × 10^7^ cells) than the control group (3.2 × 10^7^ cells). Additionally, there was no significant difference between the treatment group and sound teeth (0.2 × 10^7^ cells) that were not exposed.

### Histological observation

**Figure 6:**
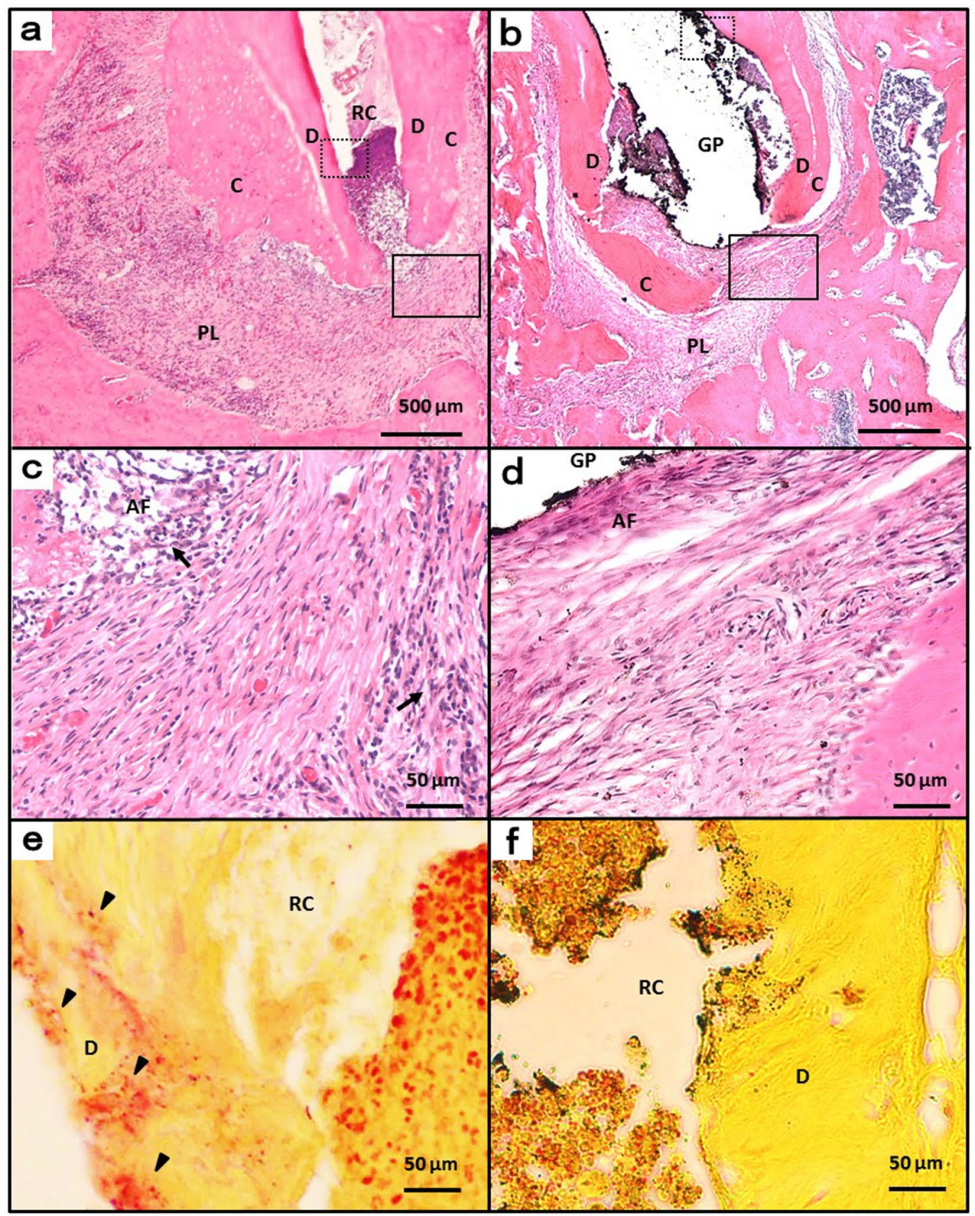
Histologic images at week 12. (**a**) Periapical areas of the control group stained with haematoxylin and eosin. (**b**) Periapical areas of the treatment group stained with haematoxylin and eosin. (**c** and **d**) High-magnification views of the solid inset in panels a and b, respectively. (**e** and **f**) High-magnification views of the dotted insets in panels b and e, respectively, stained with a modified Brown and Brenn method. AF, apical foramen; GP, gutta-percha point; RC, root canal; C, cementum; D, dentin; PL, periapical lesion; arrow head, bacteria; arrow, inflammatory cells.

In the control group, the periapical lesion contained inflammatory granulation tissue and marked inflammatory cell infiltration such as polymorphonuclear leucocytes, lymphocytes and monocytes (Fig. 6a,c). However, there was little granulation tissue in the treatment group; the area of periapical lesion decreased and normal periodontal tissue was observed in the apical region (Fig. 6b,d). Modified Brown and Brenn staining was undertaken to confirm the residue of root canal bacteria. Bacteria were observed in the root canals of the control group (Fig. 6e), but only low levels of residual bacteria were found in the root canals of the treatment group (Fig. 6f).

## Discussion

To elucidate the pathophysiology of apical periodontitis and develop therapeutic methods, pathological condition models that experimentally induce periapical lesions in animals have been widely used^18–20, 24–26^. A diverse range of animals have been used, including rats, cats, dogs and monkeys. Large numbers of rats can be studied over a short timeframe. The bacterial flora in the infected root canals of the rat differs slightly from human bacterial flora, but the ratio of aerobic bacteria to anaerobic bacteria is similar^27^, so it seems appropriate to use rats as a pathological model of apical periodontitis. However, all studies to date investigating infected root canal treatment have used large animals rather than rats^28–32^. It is difficult to establish a root canal treatment model in animals because of their small tooth size and the anatomical complexity of the root canal, and there are no studies using an animal model in which all four root canals have been treated. This is thought to be one of the causes of delays in the developmental research into the treatment and medication of infected root canals. Therefore, in this study, we tried to establish a model of infected root canal treatment using the dental microscope and other advanced equipment developed under modern technological innovation.

The crown of the rat mandibular first molar is approximately 3 mm in diameter mesiodistally, and 2 mm in diameter buccolingually, which is about one-fourth of the dimensions of the human mandibular first molar. In addition, rat teeth have four roots (buccal, lingual, mesial and distal), and the morphology is different from human teeth (Fig. 2a). Therefore, it is difficult to perform root canal treatment with the naked eye using endodontic instruments designed for human teeth. We solved these problems by using a microscope with a maximum magnification of ×32, in contrast with the usual maximum magnification of ×20–25 in microscopes used in clinical situations. This allowed us to observe and treat the inside of the pulp cavity and the root canal of rat teeth under higher magnification than is possible in normal clinical situations. Additionally, we used instruments with a tip diameter of 0.15–0.30 mm, which were small enough to be inserted into rat root canals. Given the known benefits of rubber dam isolation in root canal treatment^33, 34^, custom-made rubber dam clamps as used in previous studies^35, 36^ were also used in this study to achieve moisture-free aseptic conditions.

Some studies that used an experimental apical periodontitis model to investigate the influence of novel therapeutic drugs on the immune response used immature teeth^19^. Because it was necessary to perform complete root canal treatment in our study, we used teeth with completely developed roots, allowing us to maintain the correct working length to avoid failures such as overfilling. However, as rats become older, calcification of the teeth progresses and the root canal narrows, making it difficult to use files. Therefore, having observed the maturation process in sound rat tooth roots over time using micro-CT imaging, 10-week-old rats were chosen for use in this experiment.

In the root canal treatment model, the volume of periapical lesions in the control group tended to decrease in size after 4 weeks following the pulp exposure. This is consistent with studies that histologically measured the area of periapical lesions using Wistar rats^37, 38^. It is thought that periapical lesions are in an expanding phase until 4 weeks after pulp exposure in rats in which experimental apical periodontitis is being induced, after which time the lesions become chronic and stabilised^39, 40^. The reduction of periapical lesion volume after 4 weeks in the control group might be due to the change of the host defence reaction against bacteria at that time. The entire volume of periapical lesions in the treatment group tended to decrease compared with the control group, but a statistically significant difference was observed only at 10 weeks after pulp exposure (6 weeks after treatment). In contrast, when only the mesial root lesions were measured, there was a statistically significant difference between the treatment group and the control group at all time points after 6 weeks following pulp exposure, which is after 2 weeks following treatment. Furthermore, when only the distal root lesions were measured, no statistically significant difference was observed at any time point. These findings could be related to the complexity of the morphology of the distal root, which is relatively flattened with thinner canal walls and a large curvature. It has been reported that apical root fracture can occur in human teeth as a result of root canal enlargement or preparation with files^41, 42^. Similarly, in the thin root canal wall of the rat, micro-fracture or perforation of the root canal wall can easily occur. It therefore seems that healing of periapical lesions of the distal root was inhibited and the lesion volume did not decrease. To establish a model of root canal treatment in rats, it is necessary to minimise differences caused by operator variation and root morphology. Therefore, we considered it to be appropriate to target only the mesial root of the mandibular first molar in our study.

Removal of infected dentin in the root canal was performed by mechanical removal using endodontic files and chemical irrigation with sodium hypochlorite solution. It is important to perform minimal instrumentation in order to prevent from root fracture, and to provide a taper for efficient root canal irrigation and tight root canal filling. However, to prevent root fracture, final enlargement of the root canal was set at a maximum K-file size of #20 with a taper of 4%. In this experiment, the level of bacteria in the mesial root decreased from 3.2 × 10^7^ cells to 0.8 × 10^7^ cells by performing infected root canal treatment (Fig. 5), and the volume of the periapical lesion in the mesial root decreased (Fig. 4c). However, Brown and Brenn staining confirmed that bacteria remained near the apical foramen and the lateral branch of the main root canal in the treated group (Fig. 6f). Lateral branches are present in approximately 50% of human teeth^43^, and complete removal of bacteria with Ni-Ti rotary files or manual K-files is considered impossible^44, 45^. Therefore, in clinical practice, sterile treatment is thought to be impossible, and aseptic treatment is the current aim. If we can decrease the levels of bacteria in the root canal to a certain level and prevent new infections, inflammation of the periodontal tissue around the root apex would decrease or disappear. Although residual bacteria were observed in this experiment which observed the healing process up until 8 weeks after treatment, we believe that treatment of infected root canals targeting the rat mandibular first molar was clinically successful. However, since periapical lesions did not disappear completely throughout the experimental period of this study, further longer observation might be needed to conclude about the healing.

Bacterial DNA was detected from sound teeth in the quantitative analysis of bacteria in this study. The contamination during the extraction of teeth might explain this. It is clear that the treatment group significantly decreased the amount of bacteria in the root compared to the control group (Fig. 5), and we consider this contamination does not affect the assessment of the bacterial amount in the root because the same level of contamination should be occurred in all groups. To minimise the effect of contamination, we paid close attention not to touch the mesial root to the oral mucosa during teeth extraction. Furthermore, the surface of the root was curetted using sterilized instruments to remove contaminants as much as possible. Nevertheless, bacteria were also detected from sound teeth in this research. Disinfection of the root surface by chemical agents might be the solution for this, however we did not perform it in this research since the chemical disinfection may affect bacteria in the root through the apical foramen and change the amount of bacteria we targeted. Although the method we used in this study has a problem of contamination while processing samples, we could quantify the bacterial number in the whole root including the bacteria which were in the area we could not access by instruments and the data reflects the actual bacterial amount. Among possible methods of measuring bacteria in the root canal at the present moment, this is the most practical and useful method we believe.

In this root canal treatment model in which all four root canals were treated, the volume of the lesion in the mesial root alone reflected a more precise treatment efficacy when compared with the volume of the entire periapical lesion. This is because the roots of the rat mandibular first molar, except for the mesial root, were easily fractured or perforated. However, in our preliminary experiment, three (buccal, lingual and distal) roots except for mesial root were performed pulpotomy at the root orifice with MTA cement under the rubber-dam isolation at the same time as the pulp exposure, and root canal treatment was undertaken for only the mesial root at 4 weeks after pulp exposure. We found that there was no significant difference in the lesion volume between the treated group and the control group during whole experimental period (data not shown). These results indicate the optimal condition for root canal treatment model using 10-week-old rat mandibular first molars by inducing apical periodontitis: (1) perform root canal treatment for all four root canals under rubber dam; and (2) evaluate the periapical lesions of the mesial root only.

This study confirmed the usefulness of this root canal treatment model in rats which enabled long-term observation up until eight weeks after treatment using micro-CT imaging. Using this model, which is applied to fully developed root canals, we can perform animal experiments to test novel clinical treatment methods or medicaments including anti-biofilm agents^46, 47^, which have previously only been tested in *in vitro* studies. Additionally, by modifying this treatment model, further research can be undertaken to clarify the role of extraradicular biofilm on the pathological progression of refractory apical periodontitis, and to develop novel treatment methods for refractory apical periodontitis induced by extraradicular biofilm.
